# Carcinogenic and non-carcinogenic health risk assessment of organic compounds and heavy metals in electronic cigarettes

**DOI:** 10.1038/s41598-023-43112-y

**Published:** 2023-09-25

**Authors:** Siyuan Zhao, Xi Zhang, Junji Wang, Jianzai Lin, Deyan Cao, Meilin Zhu

**Affiliations:** 1 School of Public Health, Ningxia Medical University, No. 1160 Shengli Street, Xingqing District, Yinchuan, Ningxia China; 2 School of Basic Medical Sciences, Ningxia Medical University, No. 1160 Shengli Street, Xingqing District, Yinchuan, Ningxia China

**Keywords:** Health care, Risk factors

## Abstract

E-cigarettes are now very popular in the world. Compared to traditional cigarettes, e-cigarettes are often considered safer and healthier. However, their safety remains controversial and requires further research and regulation. In this study, we aimed to understand the possible hazards to humans of four compounds (formaldehyde, acetaldehyde, acrolein, and acetone) and seven heavy metals (arsenic, cadmium, manganese, lead, copper, nickel, and chromium) contained in e-cigarette liquids and aerosols and perform a health risk assessment. We searched PubMed, CNKI, and other databases for relevant literature to obtain data on organic compounds and heavy metals in e-cigarette liquids and aerosols, and conducted acute, chronic, and carcinogenic risk assessments of various chemicals by different exposure routes. This study showed that exposure to four organic compounds and seven heavy metals in e-cigarette aerosols and e-liquids can cause varying levels of health risks in humans through different routes, with the inhalation route posing a higher overall risk than dermal exposure and oral intake. Various chemicals at high exposure doses can produce health risks beyond the acceptable range. E-cigarette designers must improve their products by changing the composition of the e-liquid and controlling the power of the device to reduce the health effects on humans.

## Introduction

An electronic cigarette is a nicotine delivery system that consists of four main parts: cigarette oil (containing components such as nicotine, flavoring, and solvent propylene glycol), a heating system, power supply, and filter^1^. The e-liquid is heated and atomized to produce an aerosol with a specific flavor for smokers^2^. In 2003, Han Li, a pharmacist from Northeast China, invented the first nicotine-based e-cigarette product, which was popular in the market^3^. With further updates to e-cigarette manufacturing technology, a variety of models and types of e-cigarettes have entered the international market and have been welcomed by consumers in different countries^4^.

However, the use of e-cigarettes has been controversial since their invention. Some believe it is a tool to help people quit smoking, while others believe it is a new type of cigarette and can cause more harm to human health because the liquid used in e-cigarettes usually contains a mixture of propylene glycol and glycerin solvents with added nicotine and flavoring or cannabis extracts^5–11^. Propylene glycol and glycerin are almost harmless moisturizers, but when they are heated to a certain temperature, they produce harmful organic compounds such as formaldehyde, acetaldehyde, and acrolein^12^, which are classified by the International Agency for Research on Cancer (IARC) as Class 1, 2B, and 2A carcinogens, respectively^13^. A number of studies have examined the chemicals in the aerosols produced by e-cigarettes, and the results show that the majority of e-cigarette aerosols contain these organic compounds^14–18^. Electronic cigarette atomizers are generally composed of coils and wick materials, which usually contain copper, silver, zinc, tin, nickel–chromium alloys, chromium-aluminum alloys, or other metal materials^19^. Results of the substance migration measurement experiments conducted by Fan Meijuan et al.^20^.Study on metal components in e-cigarettes indicate that there is a risk of the migration of nickel and lead from metal components into aerosols. Rumasha et al.^21^ found that heavy metal elements such as tin, copper, nickel, and silicate substances were detectable in e-cigarette aerosols, and the copper content was six times higher than that in cigarette smoke, which may exacerbate DNA oxidation. Studies have shown that e-cigarettes, like traditional cigarettes, can also cause respiratory disease in humans. In July 2019, the first case of “electronic nebulizer product-associated lung disease” due to e-cigarette inhalation was reported in the United States. Since then, the number of cases has gradually increased, with thousands of people developing the disease and some resulting deaths^22^.

Accidental ingestion of e-liquid may cause acute symptoms of poisoning, and studies have shown that there is an increasing trend of acute cases due to e-liquid exposure each year^23^. An increasing number of teenagers smoke e-cigarettes, and this is considered a trend. However, several international cohort studies have shown that e-cigarette use among adolescents leads to a more than onefold increase in the likelihood of future smoking^24,25^. Teenagers are a subpopulation with hematological, nervous, endocrine, and immune systems that are still developing and may be more sensitive to the effects of toxicants. Therefore, it may pose a greater health risk^26–29^.

There are more studies on the detection of substances contained in e-cigarette liquids and aerosols but fewer on the possible risks of e-cigarettes to human health. Jefferson et al.^30^ studied the carcinogenic and non-carcinogenic risks of heavy metals contained in e-cigarette liquids and aerosols by collecting data and organizing the organs on which these heavy metals acted, and calculating the total possible risk to different organs。The results showed that the carcinogenic risk for Cr exceeded the acceptable range even at low exposure concentrations, and the non-carcinogenic risk for Ni was the highest, with an average HI of 14.5. However, in their study, they only assessed the potential harm caused by heavy metals, and the fact that organic compounds also pose health risks cannot be ignored. Vincent et al.^31^ collected data on microbial and organic compounds in e-liquids to assess the toxicity of e-liquids and showed that all products reviewed did not completely exclude potentially toxic compounds. The study was evaluated with the assumption that the concentration of chemicals would remain unchanged when the e-liquid was converted to aerosols, whereas in fact the study showed that solvents for e-liquids such as propylene glycol and glycerol produce additional aldehydes during heating^17,32^ The above literature considers both oral and inhalation routes. While the above literature considers oral and inhalation routes, the fact is that e-cigarette liquids are also hazardous for dermal contact. To clarify the degree of harm caused by the chemical components contained in e-cigarettes, we searched PubMed, CNKI, and other databases and summarized the domestic and international research data on seven heavy metal elements (arsenic, chromium, cadmium, manganese, lead, copper, and nickel) and four compounds (formaldehyde, acetaldehyde, acrolein, and acetone) produced by e-cigarettes. We used the Reference Concentration for Inhalation Exposure (RfC) and Reference Dose for Oral Exposure (RfD) data published by the U.S. Environmental Protection Agency (USEPA)^33^ as well as the Reference Exposure Levels (RELs) and Cancer Potency Values (CPVs) published by the California Office of Environmental Health Hazard Assessment (OEHHA)^34^. The health risks for these vapers, including acute toxicity, carcinogenic, and chronic non-carcinogenic risks, were then assessed by calculating daily exposures to heavy metals and organic compounds for smokers using e-cigarettes at different exposure routes. This study collects data on the major chemical hazards in e-cigarettes, including a wide range of heavy metals and organic compounds, and assesses the possible carcinogenic risk, non-carcinogenic risk, and acute ingestion risk. We performed risk assessment through each of the three exposure pathways and assessed the uncertainty and variability in the risk assessment through probabilistic assessment to bring the results closer to the real situation.

## Methods

### Data processing

This study retrieved articles from PubMed and CNKI databases, which are internationally and nationally recognized databases, to ensure the accuracy and authority of the data sources. The years 2003.1.1–2023.1.1 were set as the start and end of the search for this study, respectively, as e-cigarettes were invented in 2003. We searched the literature on the topic of “e-cigarettes” and obtained 9416 results, and then conducted advanced searches with keywords such as “e-cigarettes,” “liquid tobacco,” “aerosols,” “heavy metals,” and “chemical substances,” and obtained a total of 41 articles on chemical substance testing with clear sample types and experimental results. To facilitate the risk assessment, we eliminated chemical substances that had less data and were harmless to humans (e.g., propylene glycol and glycerol), and finally identified 28 articles with four organic compounds (formaldehyde, acetaldehyde, acrolein, and acetone) and seven heavy metal elements (arsenic, chromium, manganese, copper, lead, nickel, and cadmium) for data collection and processing. Most of these articles used laboratory smoke machines to obtain aerosols, and a few used data from volunteer smoking. These articles basically used ICP-MS and ICP-OES for detection when analyzing heavy metal elements, and a series of liquid chromatography methods including HPLC–DAD, HPLC–UV and UPLC-MS when analyzing the content of organic compounds.

We tried to use specific individual sample results from these studies for our study. If the source did not disclose the individual test results for each sample but only published the mean of the test sample results, we considered the mean a result of one sample. If only the range was published in the article, we consider the lowest and highest values as the results of two samples, respectively. If the literature studied the amount of chemicals produced by the same e-cigarette at different power levels, we calculated the average of the results detected at different power levels and consider the average as the test data for that sample. In general, chromium in the atmosphere exists as n-tris and n-hexavalent, and since the retrieved literature did not label the form of chromium obtained from the assay, we assumed that chromium in the study was the more hazardous n-hexavalent. Moreover, this study assumes that the absorption rate is 100%; i.e., the inhaled substance is completely absorbed by the body.

### Risk assessment

#### Hazard identification

The purpose of hazard identification is to recognize the presence of hazards and to characterize them. The chemicals in our study are all hazardous to humans to varying degrees. Formaldehyde and acetaldehyde are listed as carcinogens by the IARC and have different chronic non-carcinogenic effects on humans; acrolein and acetone are not carcinogenic but can be irritating to the digestive tract and internal organs and cause various inflammatory conditions if ingested continuously. Arsenic causes various inflammations, shock and even death when ingested acutely, and its chronic toxicity is manifested by damage to the human digestive tract and the carcinogenicity of inorganic arsenic. The chronic toxicity of manganese is damage to the central nervous system. Direct contact with chromium can damage the skin, respiratory tract and digestive tract, and hexavalent chromium is carcinogenic. Copper can cause gastrointestinal disturbances and other adverse effects when ingested in excess. Lead damages blood production, nerves, the digestive system, and the kidneys, and was classified as a Group 2B carcinogen in 2017. Nickel can cause dermatitis and respiratory disorders when a person ingests too much of it. Cadmium is highly toxic, causing kidney damage, emphysema and achalasia, and was classified as a group 1 carcinogen in 2017.

#### Exposure assessment

The purpose of the exposure assessment is to estimate the level of exposure of the target population to the substance to be studied. The purpose of an exposure assessment is to estimate the extent of public exposure to the emitted substance. In a study by Ayesha^23^, it was shown that accidental ingestion of e-liquids is also toxic to humans. Unintentional exposure in children and adults accounted for 87%, with intentional exposure 5% and unknown exposure < 1%. In this study, we investigated the exposure of humans to e-cigarette aerosols and e-liquids from three exposure routes: oral ingestion, inhalation, and dermal exposure. We used the acquired aerosol data to calculate exposure by the inhalation route, and the acquired e-liquid data were used to calculate exposure by oral ingestion and dermal exposure routes. For aerosols, Bertrand et al.^35^. showed in their study that the average number of daily puffs for e-cigarette users was 163. We used this value to calculate the daily exposure of vapor to organic compounds and heavy metals.

E-cigarette liquids are packaged in quantities ranging from 10 to 50 ml, and in a study by Ayesha^23^, 64.8–92.5% of people were intoxicated by ingesting whole or partially leaked e-liquids. Due to the lack of patient data on acute exposure, we assumed an acute exposure of 10 ml of e-liquid for oral and dermal exposure to calculate the acute exposure to the hazardous substance. Almost all e-cigarettes are subject to leakage failure^36^, which is mainly divided into leakage from the bottle and cigarette holder. However, different brands of e-cigarettes are made of different materials, and the habits of smokers are not the same; therefore, are no data collected on long-term exposure to direct contact with e-cigarette liquid. Daily exposure through bottle leakage is fraught with randomness, and the following factors were considered to determine a more reasonable exposure: Vincent et al.^31^ used an exposure dose of 3 g of e-liquid per day and Jefferson^30^ et al. used an exposure measure of 2 ml of e-liquid per day, which falls within the range of e-liquid reported to be consumed per day (1–10 ml/day), in calculating chronic risk. E-cigarette liquids contain glycerin and harsh-smelling additives that make it easy for the user to detect and deal with leaks if they occur, meaning that no large amounts of liquid will come into unknown contact with the skin and mouth in the event of a leak. Most users for various reasons for a small amount of leakage does not affect the use of only simple treatment, such as wiping the liquid and reassemble the device and continue to use, however, after wiping there will still be a certain amount of liquid remains on the surface of the device, and thus come into contact with the skin and the oral cavity, so we consider the exposure through leakage as unknown exposure, in the study of Ayesha^23^ the percentage of unknown exposure is less than 1%; we therefore used the assumption that 1% (0.05 ml) of the 5 ml of liquid used per day (within the range of use described above) comes into contact with the skin and oral cavity through leakage or other ways.

##### Chronic exposure

The formula for chronic daily exposure is as follows:

1 $${DD}_{inh}=\frac{{\mathrm{P}}_{\mathrm{inh}} \times \mathrm{ T }\times \mathrm{ EF }\times \mathrm{ ED}}{\mathrm{BW }\times \mathrm{ AT}}$$

2 $${DD}_{ing}=\frac{{\mathrm{P}}_{\mathrm{ing}} \times {\mathrm{CI}}_{\mathrm{ing}} \times \mathrm{ CF }\times \mathrm{ EF }\times \mathrm{ ED}}{\mathrm{BW }\times \mathrm{ AT}}$$

3 $${DD}_{derm}=\frac{{\mathrm{P}}_{\mathrm{derm}} \times {\mathrm{ CI}}_{\mathrm{derm}} \times \mathrm{ CF }\times \mathrm{ SA }\times \mathrm{ AF }\times \mathrm{ ABS }\times \mathrm{ EF }\times \mathrm{ ED}}{\mathrm{BW }\times \mathrm{ AT}}$$

*DD*_*inh*_ represents daily exposure by the inhalation route (mg/kg day), *DD*_*ing*_ represents daily exposure through oral ingestion of e-liquid (mg/kg day), and *DD*_*derm*_ represents daily exposure through dermal contact (mg/kg day). The study was based on the EPA's default exposure assumptions of a body weight of 70 kg (BW), an exposure duration of 70 years (ED), and a person inhaling 20 m^3^ of gas per day(M)^37^. Because the data units in the acquired literature are not the same, we have standardized the data for the convenience of calculation. *P* represents the concentration of chemical substances in e-cigarette aerosol and e-liquid obtained after unit harmonization; *T* represents the number of vaping sessions (163 puff)^35^, *CI* represents the assumed daily intake of e-liquid of 0.05 ml; *EF* represents the exposure frequency 365 (day/year)^37^; and *AT* represents the average exposure time, 70 × 365 days^37^; CF is the conversion factor; SA is the surface area of exposed skin, taken as 5700 cm2; AF is the skin adhesion factor 0.2 mg/(cm^2^⋅day); and ABS is the skin absorption coefficient, 0.03 for As and 0.001 for all others (unitless)^38,39^.

##### Acute exposure

The equation for acute exposure is as follows:

4 $$I=\frac{\mathrm{P}\times \mathrm{AI}}{\mathrm{BW}}$$

*P* is the concentration of the chemical in the e-liquid; *AI* is the acute intake of 10 ml^31^; and *BW* is the same as above for a body mass of 70 kg^37^.

#### Dose–response assessment

Dose–response assessment is the process of characterizing the relationship between the substance to be studied and the incidence of adverse health effects in an exposed population. In quantitative cancer risk assessment, the dose–response relationship is expressed as a slope of effectiveness and is used to calculate the probability or risk of cancer associated with the estimated exposure. In our carcinogenic risk assessment, we assumed that the risk was dose proportional and that there was no threshold for carcinogenicity, and assessed the carcinogenic risk with reference to the relevant potency slopes. For non-carcinogenic effects, we refer to chronic non-carcinogenic reference exposure levels (RELs), which are concentrations below the threshold for health effects in the general population, and compare the exposure to the threshold, and for substances exceeding the threshold we consider the risk to be unacceptable. For acute risk, we refer to the literature^31^ for the *LD*_*50*_ of a hazardous substance as its health threshold.

#### Risk characterization

This is the final step of the risk assessment. In this step, modeled concentrations and exposure information determined through exposure assessment are combined with CPVs (or SFs) and threshold concentrations (RELs or *LD*_*50*_s) developed through dose–response assessment to evaluate the carcinogenic and non-carcinogenic risks of the substances to be studied.

##### Cancer risk assessment.

As not all substances have carcinogenic risk, this study only assessed the carcinogenic risk of substances listed by the IARC^13^ or the Hot Spots Unit Risk and Cancer Potency Values from OEHHA^40^. The calculation formula is as follows:

5 $${CR}_{inh}=\mathrm{CPV}\times {\mathrm{DD}}_{\mathrm{inh}}$$

6 $${CR}_{ing}={\mathrm{SF}}_{\mathrm{ing}}\times {\mathrm{DD}}_{\mathrm{ing}}$$

7 $${CR}_{derm}={\mathrm{SF}}_{\mathrm{derm}}\times {\mathrm{DD}}_{\mathrm{derm}}$$

where *CPV* is the Cancer Potency Values published by OEHHA^40^; *DD* is the daily dose; and *SF* is the carcinogenic slope factor^41^.

According to EPA risk assessment criteria, a cancer risk of less than 1 × 10^–6^ (one additional cancer case per 1,000,000 people) is considered acceptable^42^.

##### Chronic non-carcinogenic risk assessment

We used the hazard quotient (HQ) to indicate the possible non-carcinogenic risk caused by chemical substances, which is equal to the ratio of the daily exposure to the reference level of non-carcinogenic chronic exposure^43^. Non-carcinogenic chronic exposure reference levels include RfD, REL, and RfC; however, they are derived from different agency documents^33,34^, so the reference values for the same substance in the same exposure pathway may not be equivalent. To estimate the risk from each chemical more conservatively, we compared the reference values for the same substance in the same exposure route and selected the smaller one to calculate the non-carcinogenic risk. The formula for calculating the non-carcinogenic risk is as follows:

8 $$HQ=\frac{\mathrm{NE}}{\mathrm{R}}$$

*NE* represents the daily non-carcinogenic exposure of smokers and *R* represents the reference level of non-carcinogenic chronic exposure. An HQ ≤ 1 indicates an exposure level below the reference dose with acceptable risk, an HQ > 1 indicates a possible adverse health effect, and an HQ > 10 indicates a high non-cancer risk of exposure^44^.

##### Acute risk assessment

Acute risk assessment is used to assess the risk of accidental ingestion of large amounts of e-liquid by vapers or others due to improper handling or other causes, and includes oral ingestion and dermal exposure routes. Although the US Poison Control Center receives telephone reports of acute and short-term exposures, there are very limited published data on exposure to e-cigarette aerosols and liquids^45^; therefore, this study used a presumed intake for risk assessment. The formula is as follows:

9 $$AR=\frac{\mathrm{I}}{{\mathrm{LD}}_{50}}$$

*I* is acute exposure and *LD*_*50*_ is the median lethal dose obtained from oral or dermal exposure of the chemical in animal toxicology experiments. An AR > 1 indicated that the acute risk was unacceptable.

The data required for the calculations are shown in Tables 1, 2, 3.

**Table 1:** Various reference values for assessing the risk of inhalation exposure.

	REL (μg/m^3^)^34^	CPV (mg/kg day)^-1^^40^	RfC (μg/m^3^)^33^
Formaldehyde	9	0.021	–*
Acetaldehyde	140	0.001	9
Acrolein	0.35	–	0.02
As	0.015	12	–
Cd	0.02	15	–
Mn	0.09	–	0.05
Cu	–	–	0.0402
Pb	0.5	0.042	–
Ni	0.014	0.91	–
Cr	0.2	510	0.0083

*: “–” indicates that no relevant data were found.

**Table 2:** Various reference values for assessing the risk of dermal exposure.

	RfD (mg/kg day)^33^	SF^41^	*LD*_*50*_(mg/kg)^46^
Formaldehyde	–*	–	2700
Acetone	–	–	20,000
Acrolein	–	–	562
As	0.000123	3.66	145
Cd	0.00001	6.10	–
Mn	0.00184	–	–
Cu	0.012	–	–
Pb	0.000525	0.017	–
Ni	0.0054	–	–
Cr	0.00006	20	–

*: “–” indicates that no relevant data were found.

**Table 3:** Various reference values for assessing the risk of oral exposure.

	RfD(mg/kg day)^33^	CPV (mg/kg day)^-1^^40^	*LD*_*50*_(mg/kg)^46^
Formaldehyde	0.2	–*	800
Acetaldehyde	–	–	1930
Acetone	0.9	–	5800
Acrolein	0.0005	–	46
As	0.0003	1.50	763
Cd	0.0005	0.5	–
Mn	0.14	–	–
Cu	0.04	–	–
Pb	0.0035	0.0085	–
Ni	0.02	–	–
Cr	0.003	0.42	–

*: “–” indicates that no relevant data were found.

### Uncertainty analysis

We processed the data using Crystal Ball software (Oracle Crystal Ball Enterprise Performance Management 11.1.2.4.400. 64 bit) to perform an uncertainty analysis of the relevant influences, mainly probability assessment and sensitivity analysis^47^.

#### Probability assessment

We performed the probability assessment by pre-processing data to determine the distribution of parameters (e.g., concentration of chemical substances, daily intake, frequency of exposure, and body weight). The parameter distributions were fitted using Crystal Ball software, and the best-fit probability distribution type for each variable was determined by simulating the exposure factors with Anderson–Darling and chi-square tests^48^. Probabilistic estimation of health risks was performed using Monte Carlo techniques, and the number of Monte Carlo simulations in this study was 10,000. The software randomly draws parameter values from the previously obtained distribution functions to obtain relatively stable exposure distribution results after 10,000 iterations. We used the different percentiles of exposure distribution results to assess probabilistic risk and calculate the proportion of smokers who exceed acceptable health risk levels^49^.

#### Sensitivity analysis

Sensitivity analysis was mainly used to assess the degree of contribution of each exposure factor to the results, which was also performed using Crystal Ball software. First, the rank correlation coefficients between exposure factors and health risks were determined using probability estimation methods. Subsequently, the contribution of each variable was calculated by the square of the variance^38^. Finally, the results were expressed uniformly as percentages to generate a sequence of contributing variables.

## Results

### Content of chemical substances in e-liquid and aerosols

After processing the data and summarizing the relevant literature, we obtained the results shown in Tables 4 and 5. We concluded that the mean values of formaldehyde, acetaldehyde, and acrolein in the aerosol were 0.864 μg/puff, 0.673 μg/puff, and 0.373 μg/puff, respectively. The smallest mean content was cadmium (5.63 × 10^–6^ μg/puff). The maximum formaldehyde content was 28.125 μg/puff, which was the highest among all substances. Substances other than manganese, copper, and nickel appeared to be undetected. The highest chemical content in the e-liquid was copper, with a mean value of 0.105 mg/ml and a maximum content of 0.927 mg/ml. The lowest was arsenic, which had a mean value of only 2.72 × 10^–5^ mg/ml. (See Supplementary Table S1 for data sources.)

**Table 4:** Concentration of various chemicals in aerosols (μg/puff).

	Concentration range	Mean value	Standard deviation	Number of references
Formaldehyde	0 to 2.81 × 10^1^	8.64 × 10^–1^	3.32 × 10^0^	12
Acetaldehyde	0 to 2.25 × 10^1^	6.73 × 10^–1^	2.78 × 10^0^	12
Acetone	0 to 4.11 × 10^0^	3.73 × 10^–1^	7.53 × 10^–1^	7
Acrolein	0 to 1.66 × 10^0^	2.42 × 10^–1^	3.96 × 10^–1^	5
As	0 to 1.74 × 10^–3^	2.41 × 10^–4^	3.79 × 10^–4^	3
Cd	0 to 1 × 10^–4^	5.63 × 10^–6^	1.70 × 10^–5^	6
Mn	5 × 10^–5^ to 3.21 × 10^–2^	6.75 × 10^–3^	1.08 × 10^–2^	4
Pb	0 to 2.36 × 10^–1^	3.76 × 10^–3^	2.37 × 10^–2^	8
Cu	2 × 10^–5^ to 3.84 × 10^–1^	3.47 × 10^–2^	8.05 × 10^–2^	6
Ni	1.5 × 10^–9^ to 7.38 × 10^–1^	1.47 × 10^–2^	8.99 × 10^–2^	9
Cr	0 to 1.58 × 10^–2^	3.70 × 10^–4^	1.48 × 10^–3^	9

**Table 5:** Concentration of various chemicals in e-liquid (mg/ml).

	Concentration range	Mean value	Standard Deviation	Number of references
Formaldehyde	0 to 1.97 × 10^–1^	5.41 × 10^–3^	2.08 × 10^–2^	7
Acetaldehyde	0 to 4.29 × 10^–2^	3.03 × 10^–3^	5.68 × 10^–3^	2
Acetone	0 to 1 × 10^–3^	8.61 × 10^–5^	2.16 × 10^–4^	2
Acrolein	0 to 3.27 × 10^–1^	3.06 × 10^–2^	7.04 × 10^–2^	2
As	0 to 4.30 × 10^–4^	2.72 × 10^–5^	9.27 × 10^–5^	4
Cd	1 × 10^–8^ to 2.20 × 10^–4^	7.58 × 10^–5^	5.93 × 10^–5^	6
Mn	0 to 6.91 × 10^–3^	6.35 × 10^–4^	1.79 × 10^–3^	4
Pb	0 to 1.35 × 10^–2^	5.25 × 10^–4^	1.88 × 10^–3^	7
Cu	0 to 9.27 × 10^–1^	1.05 × 10^–1^	2.41 × 10^–1^	5
Ni	0 to 6.13 × 10^–2^	3.93 × 10^–3^	1.10 × 10^–2^	6
Cr	0 to 2.11 × 10^–3^	1.34 × 10^–4^	3.29 × 10^–4^	8

### Exposure assessment and health risk assessment

#### Exposure assessment

We evaluated the exposure to various substances in the three pathways, and the results are shown in Table 6. We found that the highest average daily exposure in the inhalation route was formaldehyde (2.01 × 10^-3^ mg/kg day) , and the lowest was Cd (1.31 × 10^-8^ mg/kg day). Overall, the exposure of aldehydes was higher than that of heavy metals.The highest mean exposure under acute exposure conditions was Cu (1.50 × 10^-2^ mg/kg), and the lowest was As (3.88×10^-6^ mg/kg); the highest mean exposure under chronic exposure conditions was Cu, and the lowest was As.

**Table 6:** Exposure of each substance in different routes.

	Estimated intakes^a^ (mg/kg day)		Acute exposures^b^ (mg/kg)		Chronic oral exposures (mg/kg day)		Chronic dermal exposures (mg/kg day)	
	Range	Average	Range	Average	Range	Average	Range	Average
Formaldehyde	0 to 6.55 × 10^–2^	2.01 × 10^–3^	0 to 2.82 × 10^–2^	7.72 × 10^–4^	0 to 1.41 × 10^–4^	3.86 × 10^–6^	0 to 4.82 × 10^–3^	1.32 × 10^–4^
Acetaldehyde	0 to 5.23 × 10^–2^	1.56 × 10^–3^	0 to 6.13 × 10^–3^	4.32 × 10^–4^	0 to 3.07 × 10^–5^	2.16 × 10^–6^	0 to 1.05 × 10^–3^	7.39 × 10^–5^
Acetone	0 to 3.86 × 10^–3^	5.63 × 10^–4^	0 to 1.43 × 10^–4^	1.23 × 10^–5^	0 to 7.14 × 10^–7^	6.15 × 10^–8^	0 to 2.44 × 10^–5^	2.10 × 10^–6^
Acrolein	0 to 9.57 × 10^–3^	8.68 × 10^–4^	0 to 4.68 × 10^–2^	4.38 × 10^–3^	0 to 2.34 × 10^–4^	2.19 × 10^–5^	0 to 8.00 × 10^–3^	7.49 × 10^–4^
As	0 to 4.06 × 10^–6^	5.62 × 10^–7^	0 to 6.14 × 10^–5^	3.88 × 10^–6^	0 to 3.07 × 10^–7^	1.94 × 10^–8^	0 to 1.05 × 10^–5^	6.63 × 10^–7^
Cd	0 to 2.33 × 10^–7^	1.31 × 10^–8^	1.43 × 10^–9^ to 3.14 × 10^–5^	1.08 × 10^–5^	7.14 × 10^–12^ to 1.57 × 10^–7^	5.41 × 10^–8^	2.44 × 10^–10^ to 5.37 × 10^–6^	1.85 × 10^–6^
Mn	1.17 × 10^–4^ to 7.48 × 10^–2^	1.57 × 10^–5^	0 to 9.87 × 10^–4^	9.07 × 10^–5^	0 to 4.94 × 10^–6^	4.53 × 10^–7^	0 to 1.69 × 10^–4^	1.55 × 10^–5^
Pb	0 to 5.49 × 10^–4^	8.76 × 10^–6^	0 to 1.93 × 10^–3^	7.50 × 10^–5^	0 to 9.64 × 10^–6^	3.75 × 10^–7^	0 to 3.30 × 10^–4^	1.27 × 10^–5^
Cu	4.66 × 10^–8^ to 8.95 × 10^–4^	8.08 × 10^–5^	0 to 1.32 × 10^–1^	1.50 × 10^–2^	0 to 6.62 × 10^–4^	7.51 × 10^–5^	0 to 2.26 × 10^–2^	2.57 × 10^–3^
Ni	3.49 × 10^–12^ to 1.72 × 10^–3^	3.42 × 10^–5^	0 to 8.76 × 10^–3^	5.61 × 10^–4^	0 to 4.38 × 10^–5^	2.81 × 10^–6^	0 to 1.50 × 10^–3^	9.61 × 10^–5^
Cr	0 to 3.68 × 10^–5^	8.62 × 10^–7^	0 to 3.01 × 10^–4^	1.92 × 10^–5^	0 to 1.51 × 10^–6^	9.59 × 10^–8^	0 to 5.16 × 10^–5^	3.28 × 10^–6^

a: exposure by inhalation route.

b: exposure by dermal route and oral route.

#### Cancer risk assessment

The CRs (Cancer Risks) for the three exposure routes are shown in Table 7. We found that the overall cancer risk from inhalation exposure was higher than that from dermal exposure and oral ingestion, which may be related to the amount of exposure. We assessed the carcinogenic risk of seven substances (formaldehyde, acetaldehyde, arsenic, cadmium, lead, nickel, and chromium) for inhalation exposure. The results showed that the highest average cancer risk was for chromium (4.39 × 10^–4^), followed by formaldehyde (4.22 × 10^–5^), nickel (3.11 × 10^–5^), acetaldehyde (1.57 × 10^–5^), and arsenic (6.74 × 10^–6^). The average cancer risk for five substances was outside the acceptable range, but the cancer risk for all seven substances was greater than 1 × 10^–6^ at high dose exposure levels. We assessed the carcinogenic risk of four heavy metal elements (arsenic, cadmium, lead, and chromium) in dermal exposure. The results showed that the highest average carcinogenic risk was also for chromium (6.65 × 10^–5^), only lead does not exceed the acceptable levels. The carcinogenic risk of all four heavy metals exceeded the acceptable range at high exposure dose levels. We analyzed the carcinogenic risk of oral exposure to arsenic, cadmium, lead, and chromium. The results showed that their carcinogenic risk ranges and average carcinogenic risks were within acceptable ranges.

**Table 7:** Cancer risk of each substance in different routes.

	Inhalation exposure		Dermal exposure		Oral ingestion	
	Range	Average	Range	Average	Range	Average
Formaldehyde	0 to 1.38 × 10^–3^	4.22 × 10^–5^	–*	–	–	–
Acetaldehyde	0 to 5.23 × 10^–4^	1.57 × 10^–5^	–	–	–	–
Acetone	–	–	–	–	–	–
Acrolein	–	–	–	–	–	–
As	0 to 4.87 × 10^–5^	6.74 × 10^–6^	0 to 3.84 × 10^–5^	2.43 × 10^–6^	0 to 4.61 × 10^–7^	2.91 × 10^–8^
Cd	0 to 3.49 × 10^–6^	1.97 × 10^–7^	1.49 × 10^–9^ to 3.28 × 10^–5^	1.13 × 10^–5^	3.57 × 10^–12^ to 7.86 × 10^–8^	2.71 × 10^–8^
Mn	–	–	–	–	–	–
Pb	0 to 2.30 × 10^–5^	3.67 × 10^–7^	0 to5.61 × 10^–6^	2.16 × 10^–7^	0 to 8.20 × 10^–8^	3.19 × 10^–9^
Cu	–	–	–	–	–	–
Ni	3.17 × 10^–12^ to 1.56 × 10^–3^	3.11 × 10^–5^	–	–	–	–
Cr	0 to 1.88 × 10^–2^	4.39 × 10^–4^	0 to 1.03 × 10^–3^	6.56 × 10^–5^	0 to 6.33 × 10^–7^	4.03 × 10^–8^

* “–” indicates that the corresponding risk assessment was not performed due to lack of corresponding reference data.

#### Non-cancer risk assessment

Summarizing the HQs for the three exposure routes, the results are shown in Table 8. We found that the HQs for acrolein (151.97), manganese (1.10), copper (7.04), and nickel (8.55) exceeded the acceptable range for inhalation exposure, with acrolein having the highest HQ and being greater than 10, indicating that acrolein may have serious human health effects. Although the HQs of formaldehyde (0.78), acetaldehyde (0.61), lead (0.07), and chromium (0.38) were < 1, they were > 1 at high dose exposure levels, which also needs further attention. The HQ for all substances in e-liquids were within the acceptable range for oral ingestion exposure (0–1.66 × 10^–2^). Of the dermal exposure routes, only the HQ for copper(0–1.83 × 10^0^) at high exposure dose levels exceeded acceptable ranges.

**Table 8:** HQs of each substance in different routes.

	Inhalation exposure		Dermal exposure		Oral ingestion	
	Range	Average	Range	Average	Range	Average
Formaldehyde	0 to 2.55 × 10^1^	7.82 × 10^–1^	–*	–	0 to 7.04 × 10^–4^	1.93 × 10^–5^
Acetaldehyde	0 to 2.04 × 10^1^	6.09 × 10^–1^	–	–	–	–
Acetone	–	–	–	–	0 to 1.43 × 10^–3^	1.23 × 10^–4^
Acrolein	0 to 1.67 × 10^3^	1.52 × 10^2^	–	–	0 to 2.60 × 10^–4^	2.43 × 10^–5^
As	0 to 9.47 × 10^–1^	1.31 × 10^–1^	0 to 8.54 × 10^–2^	5.39 × 10^–3^	0 to 1.02 × 10^–3^	6.46 × 10^–5^
Cd	0 to 4.08 × 10^–2^	2.29 × 10^–3^	2.44 × 10^–5^ to 5.37 × 10^–1^	1.85 × 10^–1^	1.43 × 10^–8^ to 3.14 × 10^–4^	1.08 × 10^–4^
Mn	8.15 × 10^–3^ to 5.24 × 10^0^	1.10 × 10^0^	0 to 9.18 × 10^–2^	8.42 × 10^–3^	0 to 3.53 × 10^–5^	3.24 × 10^–6^
Pb	0 to 3.84 × 10^0^	6.59 × 10^–2^	0 to 6.29 × 10^–1^	2.42 × 10^–2^	0 to 2.76 × 10^–3^	1.07 × 10^–4^
Cu	4.06 × 10^–3^ to 7.79 × 10^1^	7.04 × 10^0^	0 to1.83 × 10^0^	2.14 × 10^–1^	0 to 1.66 × 10^–2^	1.88 × 10^–3^
Ni	8.73 × 10^–7^ to 4.29 × 10^2^	8.55 × 10^0^	0 to 2.78 × 10^–1^	1.78 × 10^–2^	0 to 2.19 × 10^–3^	1.40 × 10^–4^
Cr	0 to 1.61 × 10^1^	3.77 × 10^–1^	0 to 8.60 × 10^–1^	5.47 × 10^–2^	0 to 5.02 × 10^–4^	3.20 × 10^–5^

* “–” indicates that the corresponding risk assessment was not performed due to lack of corresponding reference data.

#### Acute risk assessment

We evaluated the acute risk of four substances (formaldehyde, acrolein, acetone, and As) via the dermal exposure route and five substances (formaldehyde, acetaldehyde, acetone, acrolein, and As) via the oral ingestion route. Owing to the lack of reference data for the remaining substances, we did not conduct an acute risk assessment. For the evaluation, we referred to the study by Vincent et al.^35^ and compared the acute exposure with *LD*_*50*_ of chemical substances. The results are shown in Table 9.We found that in the dermal exposure route, the average acute risks for the four substances were formaldehyde (2.86 × 10^-7^), acrolein (2.19 × 10^-7^), As (2.68 × 10^-8^), and acetone (2.19 × 10^-8^) in descending order. In the oral ingestion route, the average acute risks for five substances were formaldehyde (9.65 × 10^-7^), acrolein (7.55 × 10^-7^), acetone (2.67 × 10^-7^), acetaldehyde (2.24 × 10^-7^), and arsenic (5.08 × 10^-9^). The average acute exposure to each substance was very far from their *LD*_*50*_.

**Table 9:** Acute risks of each substance in different routes.

	Dermal exposure			Oral ingestion		
	Range	Average	*LD*_*50*_ (mg/kg)	Range	Average	*LD*_*50*_ (mg/kg)
Formaldehyde	0 to 1.04 × 10^–5^	2.86 × 10^–7^	2 700	0 to 3.52 × 10^–5^	9.65 × 10^–7^	800
Acetaldehyde	–*	–	–	0 to 3.18 × 10^–6^	2.24 × 10^–7^	1930
Acetone	0 to 2.54 × 10^–7^	2.19 × 10^–8^	20,000	0 to 3.11 × 10^–6^	2.67 × 10^–7^	5800
Acrolein	0 to 2.34 × 10^–6^	2.19 × 10^–7^	562	0 to 8.07 × 10^–6^	7.55 × 10^–7^	46
As	0 to 4.24 × 10^–7^	2.68 × 10^–8^	145	0 to 8.05 × 10^–8^	5.08 × 10^–9^	763

*: “–” indicates that no relevant data were found, therefore no corresponding risk assessment was carried out.

#### Total risk analysis

Because of the complexity of the composition of e-liquids and aerosols, we did not take into account synergistic or antagonistic effects between substances in calculating the total risk, but simply added up the independent risks of various substances, and the final result will be somewhat different from the actual situation. We summed the HQs, cancer and acute risks of the various routes to perform the analysis, and the results are presented in Table 10. The mean HI (sum of HQ) for inhalation exposure was 170.63, with acrolein contributing the vast majority. The mean HI for skin exposure was 5.10 × 10^–1^, and the mean HI for oral ingestion was 2.50 × 10^–3^. The HI values for both routes of exposure to e-liquid were within acceptable ranges. The average total CR for inhalation exposure was 5.35 × 10^–4^. This means that an additional 535 cases of cancer were caused by vaping in 1,000,000 people. The average total CR for dermal exposure was 7.95 × 10^–5^, and the average total CR for oral ingestion was 9.97 × 10^–8^. The total risk of cancer from the inhalation and dermal routes exceeds the acceptable range. The inhalation route poses the highest health risk, followed by dermal contact, with oral intake posing the lowest health risk. The average total Acute Risk (AR) of dermal exposure was 5.53 × 10^–7^ and the average total AR of oral ingestion was 2.22 × 10^–6^. CR and HQ from the inhalation route accounted for a large proportion of the total risk (99.70% and 87.05%, respectively). The proportion of acute risk due to oral ingestion is higher than that due to dermal exposure. The risks associated with the 11 compounds mentioned in this study contained in e-liquids are minimal during the acute ingestion of e-liquids. We calculated various risks for worst-case exposure scenarios and showed that when smokers were exposed to the highest concentrations of the compounds, the carcinogenic and non-carcinogenic risks via inhalation and dermal contact were very high.

**Table 10:** Average healthy risks for various exposure routes.

	Inhalation exposure	Dermal exposure	Oral ingestion
Average HI	170.63 (99.70%)	5.10 × 10^–1^ (0.29%)	2.50 × 10^–3^ (0.01%)
Average total CR	5.35 × 10^–4^ (87.05%)	7.95 × 10^–5^ (12.94%)	9.97 × 10^–8^ (0.01%)
Average total AR	–*	5.53 × 10^–7^ (19.95%)	2.22 × 10^–6^ (80.05%)
Maximum HI	745.97	4.3112	2.58 × 10^–2^
Maximum total CR	2.28 × 10^–2^	1.11 × 10^–3^	1.25 × 10^–6^
Maximum total AR	–*	1.34 × 10^–5^	4.96 × 10^–5^

*Because smokers rarely ingest very large amounts of e-cigarette aerosols in a short period of time, we did not do an acute risk assessment for the inhalation route.

#### Probability assessment

The results of the probability assessment in the inhalation route are shown in Table 11, where the probability of exceedance represents the probability that a smoker will be exposed to a health hazard owing to excessive chemical substance involvement. The highest exceedance probability was for acrolein (99.94%), indicating that almost all smokers were exposed to health hazards due to excessive acrolein intake. The lowest probability of exceedance was for Cd (0.01%), and the probability of exceedance for As (0.90%) and Pb (1.20%) was also very low, indicating that these three substances do not contribute much to the health risk. The probability of exceedance for Cu (77.57%) and Ni (56.97%) is more than 50% and needs to be taken seriously. The HI for dermal exposure and the oral route was very small; therefore, we did not perform an uncertainty analysis.

**Table 11:** Certainty level and exceedance probability of chemicals by the inhalation route.

Chemicals	Certainty (%)	Exceedance probability (%)
Formaldehyde	83.49	16.51
Acetaldehyde	87.45	12.55
Acrolein	0.06	99.94
As	99.10	0.90
Cd	99.99	0.01
Mn	68.77	31.23
Pb	98.80	1.20
Cu	22.43	77.57
Ni	43.03	56.97
Cr	92.77	7.23

*1—certainty = probability of exceedance. The specific results are shown in Supplementary Figs. S1 to S10.

We characterized the contribution of each parameter to the results using sensitivity analysis. Regarding the mean carcinogenic risk of the inhalation route, the concentration of chromium was the highest contributor to the total carcinogenic risk (87.9%), followed by formaldehyde (5.6%) and nickel (3.0%). In terms of the mean HI of the inhalation route, acrolein emerged as the highest contributor to the total non-carcinogenic risk (96.0%), followed by copper (1.2%). This indicates that inhalation of acrolein is closely related to non-carcinogenic risk.

Since the non-carcinogenic risks of dermal exposure and oral ingestion were very small, we only performed a sensitivity analysis for the carcinogenic risks of these two routes. The highest contribution to the mean total carcinogenic risk in the dermal exposure route was the concentration of Cr (89.4%), followed by Cd (5.7%) and As (1.4%). The highest contribution to the mean total carcinogenic risk in oral ingestion was the concentration of chromium (46.7%), followed by As (28.6%) and Cd (18.0%). This suggests that the impact of chromium, arsenic, and cadmium intake on cancer risk is not negligible.

## Discussion

This risk assessment found that smoking e-cigarettes was an important pathway for human exposure to aldehydes, ketones, and heavy metals. The average carcinogenic risks of formaldehyde, acetaldehyde, chromium, and nickel contained in the aerosols produced by e-cigarettes exceeded the acceptable range, and the risk would be even higher if the carcinogenic risks of all chemicals were added together to assess the actual harm to humans. Compared to traditional cigarettes, smoking e-cigarettes not only causes excessive intake of carcinogenic aldehydes (such as formaldehyde and acetaldehyde), but also excessive intake of heavy metals. Among the seven heavy metals studied, Cr and Ni were the main carcinogenic heavy metals, and their excessive levels were factors affecting the risk of cancer. The presence of high levels of Cr and Ni in e-cigarette aerosols may be related to the migration of heavy metals from the metal components of e-cigarettes. Meijuan et al.^20^ experimentally found the risk of migration of nickel from heating wires to aerosols and lead migration from metal parts in contact with e-liquid/aerosols, mainly from the migration of impure lead in copper-zinc alloys. This study supports this idea. The study also indicates that there is no risk of heavy metal migration from metal parts in contact with the oral cavity; therefore, only the heavy metals originally contained in e-liquids were considered in our study when assessing oral exposure.

In the chronic non-carcinogenic risk assessment, we found that the main risk of inhalation exposure originated from excessive acrolein intake. Although acrolein was classified as a group 2A carcinogen by the IARC in 2020, there are few data on its carcinogenic effects in humans, and the IARC considers the evidence on human cancers to be “inadequate,” so only the non-carcinogenic risk of acrolein was considered in this study. The non-carcinogenic risk of acrolein is very high compared to other organic compounds, with acrolein contributing 151.97 of the assessed mean HI (170.63). By comparing the values of acrolein in the liquid and aerosol, we found that although the liquid also contains acrolein, the content of acrolein in the aerosol is much larger than in the liquid, so we inferred that excess acrolein in the aerosol may come from the heating of glycerin in the cigarette liquid. Its boiling point is only 52.5 °C; hence, it is easy to produce a large amount of acrolein in the process of heating the liquid by human inhalation. The non-carcinogenic risk caused by heavy metal exposure in the inhalation route is mainly derived from manganese, copper, and nickel, which may also be related to the migration of heavy metals from e-cigarettes, as mentioned above. This is because e-cigarette heating wires are made of different materials. The heating wire of the e-cigarette system used by Williams et al.^50^ in their study was a nickel–chromium wire connected to a thicker silver-plated copper wire. They found green deposits containing copper in the fibers of both dust collectors and aerosols containing heavy metal particles with a diameter of 1 mm. The hazards associated with these particles entering alveolar cells are much greater than those caused by direct human contact with e-liquids.

The amount of chemicals in the aerosols produced by e-cigarettes is highly variable and is related to many factors. In their study, Olmedo et al.^51^ evaluated the effects of e-cigarette power, resistance, and frequency of coil replacement on the variation of heavy metal content in aerosols, showing that the Al, Co, Pb, and Zn content decreased with increasing power, while Cu, Mn, Ni, Sb, and Sn content were highest at medium power. Kosmider et al. ^17^ found in their study that e-liquids with different nicotine solvents had different carbonyl levels in aerosols produced after heating. Overall, The two main solvents for e-liquids are PG (propylene glycol) and VG (vegetable glycerine). The PG-based e-liquid produced significantly higher carbonyl group levels than the VG-based e-liquid (P < 0.05). The higher the output voltage of the battery, the higher the carbonyl content of the vapor. Gillman et al.^18^ investigated the effect of variable power levels on total aerosol mass production and aldehyde formation in e-cigarettes, and the results obtained showed that the total mass of aerosol produced by e-cigarettes gradually increased with increasing voltage, which was also accompanied by more aldehyde production. The above findings suggest that we should also consider factors such as device power and liquid composition when assessing the health risks of e-cigarettes, but they also show that the level of chemical hazards produced by e-cigarette products is controllable.

Although we assessed the acute and chronic risks (including cancer risks) that may be associated with exposure to e-liquids, the factors influencing these risks are highly variable. First, the composition of each brand of e-liquid varies, and second, there is no clear level of exposure to e-liquid for dermal and oral exposure. Varlet et al.^31^ made assumptions about e-liquid exposure in their risk assessment study (10 ml for acute exposure and 3 g/day for chronic exposure). Our assessment was informed by the acute exposures they assumed in their study and by the comparison of acute exposures to the *LD*_*50*_ of the chemical to assess acute risk, but did not use the chronic exposures they assumed. They viewed chronic exposure to e-liquid as its conversion to aerosol in their study and assumed that the composition of the liquid would not change upon heating and evaporation during e-cigarette use, as well as assuming that the concentration of the chemical in the aerosol was similar to the concentration in the e-cigarette liquid. However, these assumptions were not necessarily confirmed. The hypothesis of this study for liquid ingestion through the skin and mouth is based on the setting of “leakage,” i.e., direct contact between the human body and e-liquid, which is highly dependent on the quality of the e-cigarette. In the survey, it was found that, owing to the cost, many people deal with leaks by drying the leaking liquid and continuing to use the device, and choosing only to replace the equipment whose leaks are affecting its use, which invariably increases the amount of dermal exposure. As for oral exposure, because e-liquid has a strong irritating and unpleasant taste, a small amount of leakage will enable people to detect it in time, and leakage from vaping does not occur daily because people tend to replace devices that always fail, which will result in a significant reduction in oral exposure. Although most e-cigarettes have a factory pass rate of 99% or more, the service life of e-cigarettes varies for each brand and model, and the assumptions we've made so far are not the most rigorous due to a lack of research data, and it is expected that there will be follow-up research results that will make it more complete. It is worth noting that although the acute risk results assessed in this study were low, this does not mean that there is no risk of acute ingestion of e-liquid. In fact, e-liquid is a very complex mixture, which in addition to the mentioned organic compounds and heavy metal elements, also contains flavor, nicotine, and other compounds. These substances alone may not seem toxic, but perhaps there is a synergistic effect of undiscovered hazardous substances, so that the actual safety risk is greater than expected.

## Conclusion

We assessed the potential acute, chronic, and carcinogenic risks associated with e-cigarette products through three routes: inhalation, dermal exposure, and oral ingestion. We found that inhalation was the primary route of exposure to e-cigarette hazards. Further, the health risks of almost all substances were exceeded at high exposure dose levels, with exposure to chemical substances being an important influencing factor. This shows that excessive e-cigarette smoking can indeed bring about many hazards, which is inconsistent with the popularly believed consumer notion that smoking e-cigarettes does not affect their health. This study also illustrates that the hazards associated with e-cigarettes can be controlled; we expect that future products will be designed to minimize health risks by changing the composition of the e-liquid and controlling the power of the device.

There is still a lack of research on the health assessment of e-cigarettes, and in order to improve the risk assessment, we need to consider the possible synergistic or antagonistic effects of various chemicals when assessing the risk, the comparisons between various brands and types of e-cigarettes (liquid composition, heating rate, flavor content, etc.), and the influence of e-cigarette users' habits on the risk of health, among other things, in the following research.

### Supplementary Information

Supplementary Information 1.

Supplementary Information 2.

## Data Availability

The datasets generated during and/or analysed during the current study are available from the corresponding author on reasonable request.

## Abbreviations

ABS: Dermal absorption factor
AF: Skin adherence factor of soil
AR: Acute risk
AT: Average exposure time
BW: Body weight of exposed individual
CF: Conversion factor
CPVs: Cancer Potency Values
CR: Cancer risks
DD: Daily dose
ED: Exposure duration
EF: Exposure frequency
HI: Hazard index
HPLC–DAD: High-performance liquid chromatography-diode array detection
HPLC–UV: High-performance liquid chromatography-ultraviolet
HQ: Hazard quotient
IARC: The International Agency for Research on Cancer
ICP-MS: Inductively coupled plasma mass spectroscopy
ICP-OES: Inductively Coupled Plasma Optical Emission Spectrometer
NE: Daily Non-carcinogenic exposure
OEHHA: The California Office of Environmental Health Hazard Assessment
PG: Propylene glycol
RELs: Reference Exposure Levels
RfC: Reference Concentration
RfD: Reference Dose
SA: Surface area of exposed skin
SF: Carcinogenic slope factor
USEPA: The U.S. Environmental Protection Agency
UPLC-MS: Ultra-high performance liquid chromatography-tandem mass spectrometry
VG: Vegetable glycerine
