# Supplementary material for: Carcinogenic and non-carcinogenic health risk assessment of organic compounds and heavy metals in electronic cigarettes
Source: Sci Rep. 2023 Sep 25;13:16046. doi: 10.1038/s41598-023-43112-y (PMC10520052; doi:10.1038/s41598-023-43112-y)
Supplement: Supplementary file 1 — Supplementary Information 1. [file 41598_2023_43112_MOESM1_ESM.docx]

Carcinogenic and Non-Carcinogenic Health Risk Assessment of Organic Compounds and Heavy Metals in Electronic Cigarettes

Supplementary Information 1

Data and source literature:

Supplementary Table S1.Chemical content in e-cigarette aerosols

|  | Concentration range | Mean value | Standard Deviation | Data sources |
| --- | --- | --- | --- | --- |
| Formaldehyde | 0 to 2.81×10^1^ | 8.64×10^-1^ | 3.32×10^0^ | [1]-[10][27][28] |
| Acetaldehyde | 0 to 2.25×10^1^ | 6.73×10^-1^ | 2.78×10^0^ | [1]-[10][27][28] |
| Acetone | 0 to 4.11×10^0^ | 3.73×10^-1^ | 7.53×10^-1^ | [2][3][5][7]-[10] |
| Acrolein | 0 to 1.66×10^0^ | 2.42×10^-1^ | 3.96×10^-1^ | [1] [4]-[6][28] |
| As | 0 to 1.74×10^-3^ | 2.41×10^-4^ | 3.79×10^-4^ | [1][2][26] |
| Cd | 0 to 1×10^-4^ | 5.63×10^-6^ | 1.70×10^-5^ | [1][4][11][13][14][26] |
| Mn | 5×10^-5^ to 3.21×10^-2^ | 6.75×10^-3^ | 1.08×10^-2^ | [1][11][25][26] |
| Pb | 0 to 2.36×10^-1^ | 3.76×10^-3^ | 2.37×10^-2^ | [2][4][5][12]-[16] |
| Cu | 2×10^-5^ to 3.84×10^-1^ | 3.47×10^-2^ | 8.05×10^-2^ | [1][11][14][15][25][26] |
| Ni | 1.5×10^-9^ to 7.38×10^-1^ | 1.47×10^-2^ | 8.99×10^-2^ | [1][7][11]-[15][25][26] |
| Cr | 0 to 1.58×10^-2^ | 3.70×10^-4^ | 1.48×10^-3^ | [1][4][7][11]-[15][26] |

Supplementary Table S2.Chemical content in e-liquid

|  | Concentration range | | Mean value | Standard Deviation | Data sources |
| --- | --- | --- | --- | --- | --- |
| Formaldehyde | 0 to 1.97×10^-1^ | 5.41×10^-3^ | | 2.08×10^-2^ | [1][10][16]-[19][24] |
| Acetaldehyde | 0 to 4.29×10^-2^ | 3.03×10^-3^ | | 5.68×10^-3^ | [16][24] |
| Acetone | 0 to 1×10^-3^ | 8.61×10^-5^ | | 2.16×10^-4^ | [1][18] |
| Acrolein | 0 to 3.27×10^-1^ | 3.06×10^-2^ | | 7.04×10^-2^ | [10][18] |
| As | 0 to 4.30×10^-4^ | 2.72×10^-5^ | | 9.27×10^-5^ | [1][20][21][26] |
| Cd | 1×10^-8^ to 2.20×10^-4^ | 7.58×10^-5^ | | 5.93×10^-5^ | [1][4][15][20][22][23] |
| Mn | 0 to 6.91×10^-3^ | 6.35×10^-4^ | | 1.79×10^-3^ | [1][20][22][26] |
| Pb | 0 to 1.35×10^-2^ | 5.25×10^-4^ | | 1.88×10^-3^ | [1][15][20]-[23][26] |
| Cu | 0 to 9.27×10^-1^ | 1.05×10^-1^ | | 2.41×10^-1^ | [1][15][20][23][26] |
| Ni | 0 to 6.13×10^-2^ | 3.93×10^-3^ | | 1.10×10^-2^ | [1][15][20][22][23][26] |
| Cr | 0 to 2.11×10^-3^ | 1.34×10^-4^ | | 3.29×10^-4^ | [1][5][15][20]-[23][26] |

Reference:

[1] W. Visser et al. The health risks of using e-cigarettes. National Institute for Public Health and the Environment. Ministry of Heath, Welfare and Sport. RIVM Letter report 2015-0144.

[2] Talih S, Salman R, El-Hage R, Karam E, Salam S, Karaoghlanian N, El-Hellani A, Saliba N, Shihadeh A. A comparison of the electrical characteristics, liquid composition, and toxicant emissions of JUUL USA and JUUL UK e-cigarettes. Sci Rep. 2020 Apr 30;10(1):7322. doi: 10.1038/s41598-020-64414-5. PMID: 32355323; PMCID: PMC7192936.

[3] Kosmider L, Kimber CF, Kurek J, Corcoran O, Dawkins LE. Compensatory Puffing With Lower Nicotine Concentration E-liquids Increases Carbonyl Exposure in E-cigarette Aerosols. Nicotine Tob Res. 2018 Jul 9;20(8):998-1003. doi: 10.1093/ntr/ntx162. PMID: 29065196.

[4] Beauval N, Antherieu S, Soyez M, Gengler N, Grova N, Howsam M, Hardy EM, Fischer M, Appenzeller BMR, Goossens JF, Allorge D, Garçon G, Lo-Guidice JM, Garat A. Chemical Evaluation of Electronic Cigarettes: Multicomponent Analysis of Liquid Refills and their Corresponding Aerosols. J Anal Toxicol. 2017 Oct 1;41(8):670-678. doi: 10.1093/jat/bkx054. PMID: 28985322.

[5] Flora JW, Wilkinson CT, Wilkinson JW, Lipowicz PJ, Skapars JA, Anderson A, Miller JH. Method for the Determination of Carbonyl Compounds in E-Cigarette Aerosols. J Chromatogr Sci. 2017 Feb;55(2):142-148. doi: 10.1093/chromsci/bmw157. Epub 2016 Oct 7. PMID: 28087758; PMCID: PMC5253970.

[6] Bekki K, Uchiyama S, Ohta K, Inaba Y, Nakagome H, Kunugita N. Carbonyl compounds generated from electronic cigarettes. Int J Environ Res Public Health. 2014 Oct 28;11(11):11192-200. doi: 10.3390/ijerph111111192. PMID: 25353061; PMCID: PMC4245608.

[7] Cunningham A, McAdam K, Thissen J, Digard H. The Evolving E-cigarette: Comparative Chemical Analyses of E-cigarette Vapor and Cigarette Smoke. Front Toxicol. 2020 Dec 15;2:586674. doi: 10.3389/ftox.2020.586674. PMID: 35296117; PMCID: PMC8915913.

[8] Jo SH, Kim KH. Development of a sampling method for carbonyl compounds released due to the use of electronic cigarettes and quantitation of their conversion from liquid to aerosol. J Chromatogr A. 2016 Jan 15;1429:369-73. doi: 10.1016/j.chroma.2015.12.061. Epub 2015 Dec 23. PMID: 26748866.

[9] Talih S, Salman R, El-Hage R, Karam E, Karaoghlanian N, El-Hellani A, Saliba N, Shihadeh A. Characteristics and toxicant emissions of JUUL electronic cigarettes. Tob Control. 2019 Nov;28(6):678-680. doi: 10.1136/tobaccocontrol-2018-054616. Epub 2019 Feb 11. PMID: 30745326; PMCID: PMC7341718.

[10] Reilly SM, Bitzer ZT, Goel R, Trushin N, Richie JP. Free Radical, Carbonyl, and Nicotine Levels Produced by Juul Electronic Cigarettes. Nicotine Tob Res. 2019 Aug 19;21(9):1274-1278. doi: 10.1093/ntr/nty221. PMID: 30346584; PMCID: PMC7182768.

[11] Zhao D, Ilievski V, Slavkovich V, Olmedo P, Domingo-Relloso A, Rule AM, Kleiman NJ, Navas-Acien A, Hilpert M. Effects of e-liquid flavor, nicotine content, and puff duration on metal emissions from electronic cigarettes. Environ Res. 2022 Mar;204(Pt C):112270. doi: 10.1016/j.envres.2021.112270. Epub 2021 Oct 27. PMID: 34717948; PMCID: PMC9140018.

[12] Lu Yifeng, Li Yongxia, Zhang Hunchi et al. Simultaneous determination of seven heavy metals in e-cigarette aerosol by inductively coupled plasma mass spectrometry[J]. Journal of Analytical Testing,2020,39(06):729-735.

[13] Ting CY, Ahmad Sabri NA, Tiong LL, Zailani H, Wong LP, Agha Mohammadi N, Anchah L. Heavy metals (Cr, Pb, Cd, Ni) in aerosols emitted from electronic cigarettes sold in Malaysia. J Environ Sci Health A Tox Hazard Subst Environ Eng. 2020;55(1):55-62. doi: 10.1080/10934529.2019.1665950. Epub 2019 Sep 18. PMID: 31530230.

[14] Gonzalez-Jimenez N, Gray N, Pappas RS, Halstead M, Lewis E, Valentin-Blasini L, Watson C, Blount B. Analysis of Toxic Metals in Aerosols from Devices Associated with Electronic Cigarette, or Vaping, Product Use Associated Lung Injury. Toxics. 2021 Sep 29;9(10):240. doi: 10.3390/toxics9100240. PMID: 34678936; PMCID: PMC8537407.

[15] Gray N, Halstead M, Valentin-Blasini L, Watson C, Pappas RS. Toxic Metals in Liquid and Aerosol from Pod-Type Electronic Cigarettes. J Anal Toxicol. 2022 Feb 14;46(1):69-75. doi: 10.1093/jat/bkaa185. PMID: 33270129; PMCID: PMC9531718.

[16] Chen G, She S, Zhou SH et al. Determination of eight volatile carbonyl compounds in e-cigarette liquid smoke by high performance liquid chromatography[J]. Anhui Agricultural Science,2014,42(31):11072-11074+11082.DOI:10.13989/j.cnki.0517-6611.2014.31.087.

[17] Lim,H. ,& Shin H.(2013).Measurement of Aldehydes in Replacement Liquids of Electronic Cigarettes by Headspace Gas Chromatography-mass Spectrometry. Bulletin of the Korean Chemical Society,34(),2691-2696.https://doi.org/10.5012/bkcs.2013.34.9.2691

[18] PAN LiNing, LIU Shaofeng, LI Dongliang et al. Simultaneous determination of eight carbonyl species in e-cigarette liquids by aqueous dispersion-DNPH derivatization-LC-MS/MS[J]. Tobacco Science and Technology,2021,54(08):63-70.DOI:10.16135/j.issn1002-0861.2021.0176.

[19] DU Ruyun, XU Hongbin, BIAN Hua. Determination of formaldehyde, acetaldehyde, acrolein and 2,3-butanedione in electronic fuming solution by high performance liquid chromatography[J]. Flavor & Fragrance Cosmetics,2020,No.178(01):16-20.

[20] Mara A, Langasco I, Deidda S, Caredda M, Meloni P, Deroma M, Pilo MI, Spano N, Sanna G. ICP-MS Determination of 23 Elements of Potential Health Concern in Liquids of e-Cigarettes. Method Development, Validation, and Application to 37 Real Samples. Molecules. 2021 Nov 4;26(21):6680. doi: 10.3390/molecules26216680. PMID: 34771088; PMCID: PMC8588553.

[21] LI Q-Q,LU Z-Y,CHEN G-Y. Determination of multiple heavy elements in electron smelt liquids by microwave ablation-inductively coupled plasma mass spectrometry. Analytical Instruments,2019,No.226(05):18-23.

[22] Hess CA, Olmedo P, Navas-Acien A, Goessler W, Cohen JE, Rule AM. E-cigarettes as a source of toxic and potentially carcinogenic metals. Environ Res. 2017 Jan;152:221-225. doi: 10.1016/j.envres.2016.09.026. Epub 2016 Oct 28. PMID: 27810679; PMCID: PMC5135636.

[23] Gray N, Halstead M, Gonzalez-Jimenez N, Valentin-Blasini L, Watson C, Pappas RS. Analysis of Toxic Metals in Liquid from Electronic Cigarettes. Int J Environ Res Public Health. 2019 Nov 13;16(22):4450. doi: 10.3390/ijerph16224450. PMID: 31766137; PMCID: PMC6888324.

[24] Varlet V, Farsalinos K, Augsburger M, Thomas A, Etter JF. Toxicity assessment of refill liquids for electronic cigarettes. Int J Environ Res Public Health. 2015 Apr 30;12(5):4796-815. doi: 10.3390/ijerph120504796. PMID: 25941845; PMCID: PMC4454939.

[25] Williams M, Villarreal A, Bozhilov K, Lin S, Talbot P. Metal and silicate particles including nanoparticles are present in electronic cigarette cartomizer fluid and aerosol. PLoS One. 2013;8(3):e57987. doi: 10.1371/journal.pone.0057987. Epub 2013 Mar 20. PMID: 23526962; PMCID: PMC3603976.

[26] Olmedo P, Rodrigo L, Grau-Pérez M, Hilpert M, Navas-Acién A, Téllez-Plaza M, Pla A, Gil F. Metal exposure and biomarker levels among e-cigarette users in Spain. Environ Res. 2021 Nov;202:111667. doi: 10.1016/j.envres.2021.111667. Epub 2021 Jul 10. PMID: 34256077.

[27] Kosmider L, Sobczak A, Fik M, Knysak J, Zaciera M, Kurek J, Goniewicz ML. Carbonyl compounds in electronic cigarette vapors: effects of nicotine solvent and battery output voltage. Nicotine Tob Res. 2014 Oct;16(10):1319-26. doi: 10.1093/ntr/ntu078. Epub 2014 May 15. PMID: 24832759; PMCID: PMC4838028.

[28] Gillman IG, Kistler KA, Stewart EW, Paolantonio AR. Effect of variable power levels on the yield of total aerosol mass and formation of aldehydes in e-cigarette aerosols. Regul Toxicol Pharmacol. 2016 Mar;75:58-65. doi: 10.1016/j.yrtph.2015.12.019. Epub 2015 Dec 29. PMID: 26743740.
