# Supplementary material for: Carcinogenic and non-carcinogenic health risk assessment of organic compounds and heavy metals in electronic cigarettes
Source: Sci Rep. 2023 Sep 25;13:16046. doi: 10.1038/s41598-023-43112-y (PMC10520052; doi:10.1038/s41598-023-43112-y)
Supplement: Supplementary file 2 — Supplementary Information 2. [file 41598_2023_43112_MOESM2_ESM.docx]

Carcinogenic and Non-Carcinogenic Health Risk Assessment of Organic Compounds and Heavy Metals in Electronic Cigarettes

Supplementary Information 1

Probability assessment results:

Supplementary Figure S1. Results of Probability Assessment of Formaldehyde

The level of certainty is 83.49%.

The range of certainty is from -∞ to 9.00

Supplementary Figure S2. Results of Probability Assessment of Acetaldehyde

The level of certainty is 87.45%.

The range of certainty is from -∞ to 9.00

Supplementary Figure S3. Results of Probability Assessment of Acrolein

The level of certainty is 0.06%

The range of certainty is from -∞ to 0.02

Supplementary Figure S4. Results of Probability Assessment of As

The level of certainty is 99.10%.

The range of certainty is from -∞ to 0.02

Supplementary Figure S5. Results of Probability Assessment of Cd

The level of certainty is 99.99%.

The range of certainty is from -∞ to 0.02

Supplementary Figure S6. Results of Probability Assessment of Mn

The level of certainty is 68.77%.

The range of certainty is from -∞ to 0.05

Supplementary Figure S7. Results of Probability Assessment of Pb

The level of certainty is 98.80%.

The range of certainty is from -∞ to 0.50

Supplementary Figure S8. Results of Probability Assessment of Cu

The level of certainty is 22.43%.

The range of certainty is from -∞ to 0.04

Supplementary Figure S9. Results of Probability Assessment of Ni

The level of certainty is 43.03%.

The range of certainty is from -∞ to 0.01

Supplementary Figure S10. Results of Probability Assessment of Cr

The level of certainty is 92.77%.

The range of certainty is from -∞ to 0.01
